# Stability and Robustness of Unbalanced Genetic Toggle Switches in the Presence of Scarce Resources

**DOI:** 10.3390/life11040271

**Published:** 2021-03-24

**Authors:** Chentao Yong, Andras Gyorgy

**Affiliations:** 1 Department of Chemical and Biological Engineering, New York University, New York, NY 10003, USA; yong@nyu.edu; 2 Department of Electrical and Computer Engineering, New York University Abu Dhabi, Abu Dhabi 129188, United Arab Emirates

**Keywords:** synthetic biology, competition for shared resources, modularity, toggle switch, multistability, robustness, rational design, potential landscape

## Abstract

While the vision of synthetic biology is to create complex genetic systems in a rational fashion, system-level behaviors are often perplexing due to the context-dependent dynamics of modules. One major source of context-dependence emerges due to the limited availability of shared resources, coupling the behavior of disconnected components. Motivated by the ubiquitous role of toggle switches in genetic circuits ranging from controlling cell fate differentiation to optimizing cellular performance, here we reveal how their fundamental dynamic properties are affected by competition for scarce resources. Combining a mechanistic model with nullcline-based stability analysis and potential landscape-based robustness analysis, we uncover not only the detrimental impacts of resource competition, but also how the unbalancedness of the switch further exacerbates them. While in general both of these factors undermine the performance of the switch (by pushing the dynamics toward monostability and increased sensitivity to noise), we also demonstrate that some of the unwanted effects can be alleviated by strategically optimized resource competition. Our results provide explicit guidelines for the context-aware rational design of toggle switches to mitigate our reliance on lengthy and expensive trial-and-error processes, and can be seamlessly integrated into the computer-aided synthesis of complex genetic systems.

## 1. Introduction

Living organisms synthesize a large collection of complex products relying on a vast array of intertwined processes [1]. Synthetic biology seeks to take advantage of these processes by modifying and rewiring existing connections, and via introducing novel components. This interdisciplinary field thus holds the promise of controlling cellular behavior by combining expertise from a diverse set of domains, including experimental techniques from the life sciences and quantitative tools from engineering disciplines [2,3,4]. As a result of this integrative approach, multiple industries are expected to be transformed and revolutionized, including regenerative medicine, biosensing and bioremediation, as well as sustainable manufacturing and energy production [5,6,7,8].

While synthetic biology bears many similarities to traditional engineering disciplines, designing synthetic gene circuits is often time consuming due to their context-dependent behavior [9,10,11,12,13,14], frequently leading to unexpected and perplexing phenomena [15,16,17]. Thus, the construction of even simple systems typically relies on massive DNA libraries that needs to be iteratively refined, involving high-throughput screening and testing in a lengthy and expensive process [18,19,20]. Although this library-based screening approach can prove successful for modules of modest complexity, the method rapidly becomes infeasible with increasing circuit size. Context-dependence thus poses a critical limitation in synthetic biology by undermining the modular and rational design of large-scale systems.

Unlike the above approach based on combinatorial DNA libraries, quantitative tools and computational techniques facilitate the rational forward-engineering of complex genetic circuits. This is achieved by leveraging mathematical models explicitly accounting for various sources of context-dependence, such as metabolic burden due to the limited availability of shared resources, both internal and external to organisms. As a result, synthesizing heterologous proteins can lead to growth rate reduction, and the expression of two unrelated proteins may become coupled [10,11,13,21,22,23,24,25,26,27,28,29,30]. Tackling this issue requires system-level approaches [31,32,33,34] combining a diverse set of quantitative tools [35,36,37,38,39,40,41,42,43,44,45,46,47]. This data-driven quantitative approach thus holds the promise of transforming the way complex biocircuits are designed by offering a more scalable approach [48].

Considering the central role that multistable switches play in both synthetic and natural systems with application examples ranging from regulating cell fate to autonomous control of maximizing cellular productivity [49,50,51,52,53,54,55,56,57], it is especially troubling that their behavior displays particularly strong dependence on their context [58,59]. Thus, our objective here is to reveal how tunable biophysical parameters of the toggle switch shape its fundamental properties. In particular, we seek to characterize how the interplay of competition for shared cellular resources, parameter asymmetries, and burden from the genetic context affects the stability and robustness of toggle switches [60].

Recent results illustrate that competition for shared cellular resources has profound implications regarding fundamental properties of symmetric genetic switches [61,62,63]. Here, we significantly extend these findings to the case of asymmetric toggle switches [64], thus amplifying the impact of our analysis by considering a much wider and experimentally more realistic set of circuits. Combining nullcline analysis and potential landscape-based robustness analysis, our results reveal that resource competition acts against bistability and the unbalancedness of the toggle switch further exacerbates this effect. Thus, bistability requires reduced parameter asymmetry due to resource competition, that is, the toggle switch needs to be better balanced in the presence of scarce resources [64]. Additionally, we demonstrate that both parameter asymmetries and resource competition reduce the overall robustness of metastable fixed points to noise by decreasing the potential barriers separating these equilibria. As a result, the frequency of random switching between these states increases, thus reducing the long-term reliability of the toggle switch as a memory unit. Illustrating the practical implications of our findings, we further reveal that unbalancedness significantly reduces the critical number of bistable toggle switches that can be simultaneously deployed without their collective behavior becoming monostable due to the additional resource competition they face from each other [64]. All of the above findings highlight that resource competition generally has negative impacts on the stability and robustness properties of the toggle switch. However, our analysis also reveals that by carefully adjusting resource sequestration (e.g., via the introduction of decoy sites [65]), it can also increase the balancedness of the toggle switch, an idea similar to how substrate sequestration can be leveraged for biosensor optimization [66]. As we illustrate, this is crucial for leveraging the toggle switch as a “digital comparator” in genetic optimizer modules [67].

Importantly, the results presented in this paper are underpinned by a mechanistic model capturing the scarcity of transcriptional and translational resources, leading to accurate in vivo and in vitro predictions [26,28]. By collapsing tunable microscopic model parameters (e.g., promoter strength, DNA copy number, and ribosome binding site strength) into lumped constants, we dramatically decrease complexity while preserving interpretability. Our findings thus not only provide explicit guidelines promoting modularity and increased robustness to noise, but they can also be mapped directly to experimental considerations and concrete design choices. Consequently, the stability and robustness of genetic toggle switches can be rationally adjusted by combining a wide variety of experimental tools, such as ribosome binding site and promoter engineering [20,68], introduction of decoy sites [65], and expression of heterologous proteins [25,26].

This paper is organized as follows. After briefly introducing the mathematical model of the toggle switch explicitly accounting for the limited availability of shared cellular resources, we present the quantitative framework underpinning the computational results of the paper (see Supplementary Materials for source code). Following this, we leverage these tools to reveal the role that each parameter plays in determining stability and robustness properties of the toggle switch, and how these results can be translated to explicit design guidelines for the context-aware synthesis of genetic switches.

## 2. Materials and Methods

Here, we first detail the mathematical model of the toggle switch in the presence of scarce transcriptional/translational resources. Following this, we introduce the main tools and techniques we leverage to uncover how competition for shared resources and parameter asymmetry shape the stability and robustness properties of genetic toggle switches.

### 2.1. Mathematical Model and Parameters

Comprising the repressor proteins *Y* and *Z*, the behavior of the toggle switch [60] evolves according to

(1) $$\frac{dY}{dt}=\frac{\alpha_{Y}}{1+{\left(Z/K_{Z}\right)}^{\theta_{Z}}}-\gamma_{Y}Y,\;\frac{dZ}{dt}=\frac{\alpha_{Z}}{1+{\left(Y/K_{Y}\right)}^{\theta_{Y}}}-\gamma_{Z}Z,$$

where $K_{Y}$ and $\gamma_{Y}$ are the dissociation and decay rate constants of *Y*, respectively, with Hill coefficient $\Theta_{Y}$, and $K_{Z}$, $\gamma_{Z}$, and $\Theta_{Z}$ are defined similarly. In this paper, we consider $\Theta_{Y}=\Theta_{Z}=2$ corresponding to the most commonly considered case of repressors binding as dimers [50,52,69,70,71,72,73], but our analysis can be easily extended to other cases as well [20,49,56,59,74,75]. Assuming that protein decay is primarily determined by cell growth, in this manuscript we consider $\gamma_{Y},\gamma_{Z}\approx \gamma$ where γ is the cell growth rate. Note that gratuitous protein expression can negatively affect host growth [11,21,25,76], and the extent of this effect largely depends on experimental conditions [77,78,79]. This in turn can lead to the reallocation of cellular resources [22,23,24], eventually yielding a bidirectional coupling between genetic circuits and their host, further complicating the rational analysis and design of large-scale systems. Motivated by the evidence suggesting that such effects may only be transient in the exponential phase and that they disappear after several generations of exponential growth [26,80], here, we assume that cellular growth rate is constant. In case this assumption does not hold, integrative circuit–host models [10,32,33,34,40] offer a promising avenue of inquiry to accurately predict how cell proliferation and gene expression affect one another in the above bidirectional coupling. Finally, the production rate constants $\alpha_{Y}$ and $\alpha_{Z}$ encompass all transcriptional and translational processes. For instance, considering the mechanistic model detailed in [26,28], we have that

(2) $$\alpha_{Y}=\frac{\lambda_{Y}^{\mathrm{TX}}\lambda_{Y}^{\mathrm{TL}}D}{\delta_{Y}\kappa_{Y}k_{Y}},\;\alpha_{Z}=\frac{\lambda_{Z}^{\mathrm{TX}}\lambda_{Z}^{\mathrm{TL}}D}{\delta_{Z}\kappa_{Z}k_{Z}},$$

where $\lambda^{\mathrm{TX}}$ and $\lambda^{\mathrm{TL}}$ are transcriptional and translational rate constants, respectively; κ and *k* denote the dissociation constants of RNA polymerase (RNAP) and ribosomes to their targets, respectively; and *D* and δ stand for DNA concentration and mrNA decay rate, respectively. Typical values of these parameters are provided in Table 1.

While the above model captures the dynamics of the toggle switch when transcriptional/translational resources are abundant, it fails to account for the competition phenomenon that arises when these resources are scarce [25,26,27,28]. As both repressors, as well as the genetic context of the toggle switch, rely on the same pool of resources (building blocks, energy, RNAP, ribosomes, etc.), the above coupling effects need to be modeled explicitly for predictable system-level behavior.

Accounting for the limited availability of these scarce resources, the dynamics of the toggle switch becomes

(3) $$\frac{dY}{dt}=\frac{\frac{\alpha_{Y}}{1+{\left(Z/K_{Z}\right)}^{2}}}{1+\frac{\beta_{Y}}{1+{\left(Z/K_{Z}\right)}^{2}}+\frac{\beta_{Z}}{1+{\left(Y/K_{Y}\right)}^{2}}}-\gamma Y,\;\frac{dZ}{dt}=\frac{\frac{\alpha_{Z}}{1+{\left(Y/K_{Y}\right)}^{2}}}{1+\frac{\beta_{Y}}{1+{\left(Z/K_{Z}\right)}^{2}}+\frac{\beta_{Z}}{1+{\left(Y/K_{Y}\right)}^{2}}}-\gamma Z$$

according to the works in [26,28], with the lumped constants

(4) $$\beta_{Y}=\frac{D_{Y}}{\kappa_{Y}}\left(1+\frac{\lambda_{Y}^{\mathrm{TX}}}{\delta_{Y}k_{Y}}\right),\;\beta_{Z}=\frac{D_{Z}}{\kappa_{Z}}\left(1+\frac{\lambda_{Z}^{\mathrm{TX}}}{\delta_{Z}k_{Z}}\right).$$

Note that $\beta_{Y}$ and $\beta_{Z}$ decrease the effective production rate constants of *Y* and *Z*, respectively, and this effect increases with protein production levels. Therefore, these lumped constants measure resource sequestration associated with the production of *Y* and *Z*, respectively, due to the limited availability of shared resources. For instance, $\beta_{Y}=\beta_{Z}=0$ in case of abundant resources (i.e., in the absence of competition and unwanted coupling between processes responsible for the expression of *Y* and *Z*).

Finally, to simplify further analysis, introduce the dimensionless quantities

$$y=\frac{Y}{K_{Y}},\;z=\frac{Z}{K_{Z}},\;\alpha_{y}=\frac{\alpha_{Y}}{\gamma K_{Y}},\;\alpha_{z}=\frac{\alpha_{Z}}{\gamma K_{Z}},\;t^{\prime }=\gamma t,$$

together with $\beta_{y}=\beta_{Y}$ and $\beta_{z}=\beta_{Z}$, so that (3) becomes

(5) $$\frac{dy}{dt^{\prime }}=\frac{\frac{\alpha_{y}}{1+z^{2}}}{1+\frac{\beta_{y}}{1+z^{2}}+\frac{\beta_{z}}{1+y^{2}}}-y,\;\frac{dz}{dt^{\prime }}=\frac{\frac{\alpha_{z}}{1+y^{2}}}{1+\frac{\beta_{y}}{1+z^{2}}+\frac{\beta_{z}}{1+y^{2}}}-z.$$

Based on the typical parameter ranges presented in Table 1, we estimate $\alpha_{y},\alpha_{z}\approx 1\cdots 100$ and $\beta_{y},\beta_{z}\approx 0.1\cdots 10$ to be typical from (2) and (4). Naturally, this range can be extended by varying promoter regions, ribosome binding sites, degradation tags, etc.

### 2.2. Stability Analysis

Considering the model in (1), neglecting the effects of resource competition, it was shown numerically that balancedness of the toggle switch (i.e., $\alpha_{y}\approx \alpha_{z}$) is essential for bistability [60]. To characterize this crucial feature, we introduce $a=\alpha_{y}/\alpha_{z}$ together with $\alpha_{0}=\sqrt{\alpha_{y}\alpha_{z}}$ measuring the mean expression strength of the repressors. With this, we write the dynamics (5) as

(6) $$\dot{y}=f_{y}\left(y,z\right),\;\dot{z}=f_{z}\left(y,z\right)$$

such that $\alpha_{y}=\alpha_{0}\sqrt{a}$ and $\alpha_{z}=\alpha_{0}/\sqrt{a}$. In what follows, we assume that a≥1 without loss of generality (if a≤1 then swapping *y* and *z* would result in a≥1).

**Figure 1:**
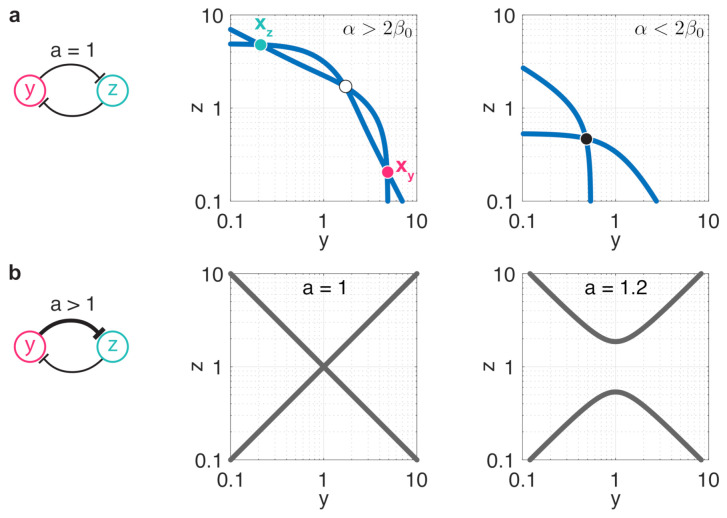
Resource sequestration and unbalancedness both act against bistability. Fixed points and stability profile are determined by the intersection of the nullclines $f_{y}\left(y,z\right)=0$ and $f_{z}\left(y,z\right)=0$ depicted in blue; stable and unstable fixed points are denoted by full and empty circles, respectively. (**a**) Stability profile in case of a balanced toggle switch (a=1). Middle panel: moderate resource sequestration ($\alpha >2\beta_{0}$) yields bistable dynamics ($\alpha_{y}=\alpha_{z}=10$, $\beta_{y}=\beta_{z}=1$). Right panel: increasing resource sequestration above the critical threshold ($2\beta_{0}>\alpha$) eventually results in monostable dynamics ($\alpha_{y}=\alpha_{z}=10$, $\beta_{y}=\beta_{z}=10$). (**b**) In the general case when a≥1, fixed points and stability profile are determined by the intersection of the nullclines with the manifold given by the constraint in (7), depicted with solid gray lines. Middle panel: in case of a balanced toggle switch we have a=1, thus (7) simplifies to y=z or yz=1. Right panel: increasing *a* pulls the two branches of (7) apart from each other, thus pushing the dynamics towards monostability.

The stability profile of (6) can be established using nullcline analysis to reveal the effects of resource competition and parameter asymmetry. Focusing first on balanced toggle switches (i.e., a=1), with $\beta_{0}=\sqrt{\left(1+\beta_{y}\right)\left(1+\beta_{z}\right)}$ the nullclines $0=f_{y}\left(y,z\right)$ and $0=f_{z}\left(y,z\right)$ intersect three times if $\alpha_{0}>2\beta_{0}$ and at a single point otherwise [61] (Figure 1a). Considering the Jacobian of (6) at these intersections, it was also shown in [61] that two of them correspond to stable fixed points, whereas the third one gives rise to an unstable equilibrium. As increased resource sequestration yields greater values of $\beta_{0}$, it pushes the nullclines lower in Figure 1a, eventually causing the transition from bistability (middle panel in Figure 1a) to monostability (right panel in Figure 1a).

While in the case of balanced toggle switches an equilibrium lies either on the y=z or on the yz=1 manifolds [61] (middle panel in Figure 1b), in case of unbalanced switches this is no longer true. In particular, fixed points of (6) given by the intersections of the nullclines $0=f_{y}\left(y,z\right)$ and $0=f_{z}\left(y,z\right)$ must also satisfy the constraint

(7) $$0=a\frac{z}{1+z^{2}}-\frac{y}{1+y^{2}},$$

as illustrated in the right panel in Figure 1b (see Appendix A for more details). As the branches of this constraint move away from each other as *a* increases, we expect unbalancedness to act against bistability. In particular, as we reveal in Section 3.1, while minor differences between $\alpha_{y}$ and $\alpha_{z}$ (i.e., a≈1) still yield bistable dynamics with stable fixed points $x_{y}$ and $x_{z}$ (*y*-dominated and *z*-dominated), exceeding a critical threshold eventually leads to a single stable fixed point, yielding monostability.

### 2.3. Robustness Analysis

To uncover how the the interplay between unbalancedness and resource sequestration affects the robustness of (6) to noise, here we study the average time trajectories spend near the metastable fixed points $x_{y}$ and $x_{z}$ before transitioning towards the other. To this end, we first extend (6) in the form of the overdamped Langevin dynamics as

(8) $$\dot{y}=f_{y}\left(y,z\right)+\sqrt{2\epsilon }\xi_{y},\;\dot{z}=f_{z}\left(y,z\right)+\sqrt{2\epsilon }\xi_{z},$$

where ϵ regulates the intensity of the zero-mean δ-correlated Gaussian white noise $(\xi_{y},$$\xi_{z})$ [63]. It is this noise that leads to trajectories (infrequently) leaving the metastable fixed points [85,86,87,88], thus causing unwanted $x_{y}↔x_{z}$ transitions. The frequency of these events can be characterized by approximating the underlying dynamics with a Markov jump process [89], where transition rates are parametrized using the Eyring–Kramers formula [85,86,87,88]. Within this framework, transition rates depend on the potential barriers separating metastable fixed points. Therefore, we next define a suitable (quasi) potential.

As $\partial f_{y}\left(y,z\right)/\partial z\neq \partial f_{z}\left(y,z\right)/\partial y$, the dynamics in (6) do not correspond to a gradient system, hence a quasi-potential must be defined [90,91]. Following one of the most common approaches, the quasi-potential V(y,z) changes along trajectories according to

(9) $$\Delta V\left(y,z\right)=-\left[f_{y}^{2}\left(y,z\right)+f_{z}^{2}\left(y,z\right)\right]\Delta t$$

for a sufficiently small time step Δt [90,92]. As ΔV(y,z)≤0 and ΔV(y,z)=0 only when $f_{y}\left(y,z\right)=f_{z}\left(y,z\right)=0$, the potential surface behaves like a Lyapunov function [90,92]: system trajectories “flow downhill” towards the metastable fixed points $x_{y}$ and $x_{z}$ (for more details on the computation of V(y,z), see Appendix B).

**Figure 2:**
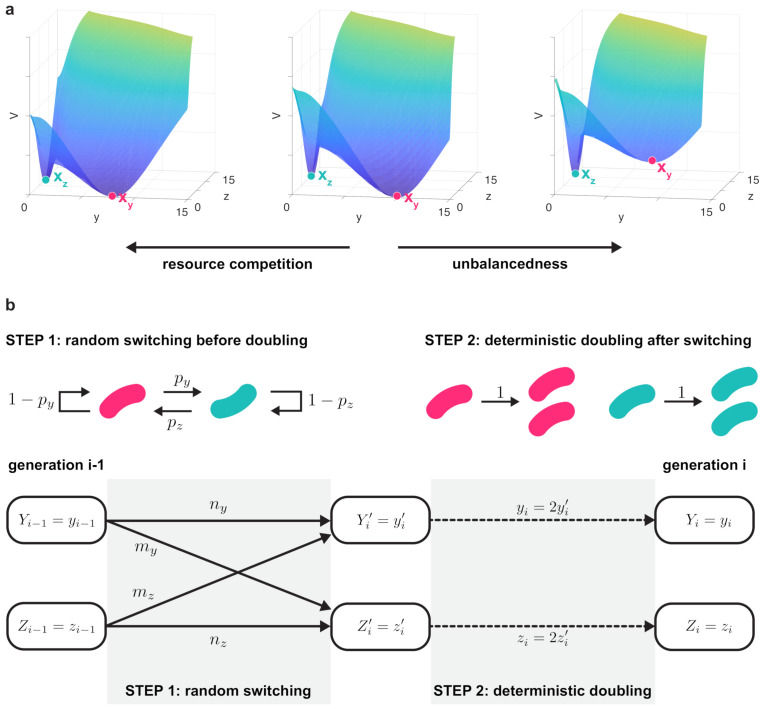
Resource competition and unbalancedness both decrease robustness to noise. (**a**) In case of balanced bistable toggle switches, the two potential barriers are identical ($h_{y}=h_{z}$, **middle panel**), and resource sequestration lowers both these potential barriers (**left panel**). Conversely, unbalancedness increases one of the potential barriers at the expense of the other (**right panel**). Simulation parameters: $\alpha_{y}=\alpha_{z}=10$, $\beta_{y}=\beta_{z}=0.25$ in the left panel; $\alpha_{y}=\alpha_{z}=10$, $\beta_{y}=\beta_{z}=0$ in the middle panel; $\alpha_{y}=9.33$, $\alpha_{z}=10.72$, $\beta_{y}=\beta_{z}=0$ in the right panel (thus a=1.15). In all panels $\alpha_{0}=10$. (**b**) Based on the robustness of the metastable fixed points, cells switch states with probabilities $p_{y}$ and $p_{z}$ (STEP 1), followed by their doubling yielding two identical cells preserving the same state (STEP 2). Before the *i*th doubling, $n_{y}$ and $n_{z}$ cells preserve their *y*-dominated and *z*-dominated states, respectively, and the rest switch states ($m_{y}$ and $m_{z}$ from the former to the latter and vice versa, respectively). The random variable $Y_{i}^{\prime }$ denotes the number of cells in the *y*-dominated state between STEP 1 and STEP 2, just before the *i*th doubling, so that $Y_{i}=2Y_{i}^{\prime }$.

According to the Eyring–Kramers formula [87,88], the average time trajectories spend near a metastable fixed point (mean transition time) is exponentially proportional to the potential barrier required for leaving its neighborhood. To characterize this time and potential barrier, let $\Omega_{y},\Omega_{z}\in R^{2}$ denote the regions of convergence of the metastable fixed points $x_{y}$ and $x_{z}$, respectively. With this, $\tau_{y}=E\left[\inf\left\{t>0:\left(y,z\right)\in \Omega_{y},\;\left(y\left(0\right),z\left(0\right)\right)\in \Omega_{z}\right\}\right]$ and $\tau_{z}=E\left[\inf\left\{t>0:\left(y,z\right)\in \Omega_{z},\;\left(y\left(0\right),z\left(0\right)\right)\in \Omega_{y}\right\}\right]$ are the average duration trajectories spend near the metastable fixed points $x_{y}$ and $x_{z}$, respectively. From the Eyring–Kramers formula, as ϵ→0 the time $\tau_{y}$ is exponentially proportional to the potential barrier $h_{y}$ required for leaving $\Omega_{y}$ (Figure 2a), defined as

(10) $$h_{y}=\underset{\gamma }{\inf}\underset{x^{*}\in \chi }{\sup}V\left(x^{*}\right)-V\left(x_{y}\right),$$

where χ denotes continuous paths leading from $x_{y}$ to $\Omega_{z}$ [85,87,88]. The potential barrier $h_{z}$ is defined similarly. Therefore, to reveal how resource sequestration and unbalancedness of the toggle switch impact its robustness to noise, in Section 3.2 we characterize how the potential barriers $h_{y}$ and $h_{z}$ separating the metastable fixed points depend on these factors.

To further understand the long-term implications of reduced robustness to noise, we consider a two-step process to model both the random transitions between the metastable states $x_{y}$ and $x_{z}$ and the doubling of cells (Figure 2b). This way it becomes possible to reveal how the population-level distribution of colonies evolve over time, and how this process is shaped by resource sequestration and unbalancedness of the toggle switch. To this end, let $p_{y}$ and $p_{z}$ denote the probability of leaving $x_{y}$ and $x_{z}$ during one cell cycle, respectively (STEP 1 in Figure 2b). Furthermore, we assume that switching happens during growth and concludes before doubling takes place, thus cells preserve their states during the deterministic doubling (STEP 2 in Figure 2b). Starting from a single cell in generation 0, the population size after *i* doublings is $N_{i}=2^{i}$.

With this, we are interested in how the population-level distribution of cell fates evolve over time, depending on the state of the seed cell in generation 0. To this end, introduce the random variables $Y_{i}$ and $Z_{i}$ to denote the number of cells in the *y*-dominated and *z*-dominated states in generation *i*, respectively. Therefore, $q_{i}\left(y_{i}\right)=\mathrm{Pr}\left(Y_{i}=y_{i}\;|\;Y_{0}=0\right)$ is the probability of observing $y_{i}$ cells in the former state after *i* doublings provided that the initial seed cell was in latter state, and similarly, let $r_{i}\left(y_{i}\right)=\mathrm{Pr}\left(Y_{i}=y_{i}\;|\;Y_{0}=1\right)$. As with this we have

(11) $$\begin{array}{cc} \mathrm{Pr}\left(Z_{i}=z_{i}\;|\;Y_{0}=0\right)= & \mathrm{Pr}\left(Y_{i}=2^{i}-z_{i}\;|\;Y_{0}=0\right)=q_{i}\left(2^{i}-z_{i}\right),\\ \mathrm{Pr}\left(Z_{i}=z_{i}\;|\;Y_{0}=1\right)= & \mathrm{Pr}\left(Y_{i}=2^{i}-z_{i}\;|\;Y_{0}=1\right)=r_{i}\left(2^{i}-z_{i}\right), \end{array}$$

the population-level composition of cell fates can be calculated by computing $q_{i}\left(\cdot \right)$ and $r_{i}\left(\cdot \right)$ for i=0,1,2,⋯, as we detail in Section 3.2.

## 3. Results

Leveraging the tools and results from the previous section, here we reveal how resource competition and parameter asymmetry affect fundamental stability and robustness properties of the toggle switch. In addition to illustrating their unwanted consequences, we also uncover how these tunable biophysical properties can be utilized, for instance, by exploiting resource sequestration to balance asymmetric toggle switches so that they can be employed in genetic optimizer modules [67]. As our approach relies on a mechanistic model of the system dynamics underpinned by biophysical parameters with clear interpretations, the findings presented here are directly translatable to experimental considerations.

### 3.1. Stability Properties

While the stability analysis of (6) is significantly more complex in the unbalanced case, it is possible to derive sufficient conditions ensuring monostability and bistability (see Appendix A for details). In particular, (6) becomes monostable if

(12) $$1+\frac{\beta_{0}}{\sqrt{b}}\geq \frac{\alpha_{0}}{\sqrt{a}+\sqrt{a-\frac{1}{a}}}.$$

In addition to providing explicit design guidelines, the above formula (together with its counterpart in Theorem A1 in Appendix A) also illuminates the role that the parameters $\alpha_{0}=\sqrt{\alpha_{y}\alpha_{z}}$, $a=\alpha_{y}/\alpha_{z}$, $\beta_{0}=\sqrt{\left(1+\beta_{y}\right)\left(1+\beta_{z}\right)}$, and $b=\left(1+\beta_{y}\right)/\left(1+\beta_{z}\right)$ play in shaping the stability profile of unbalanced toggle switches.

**Figure 3:**
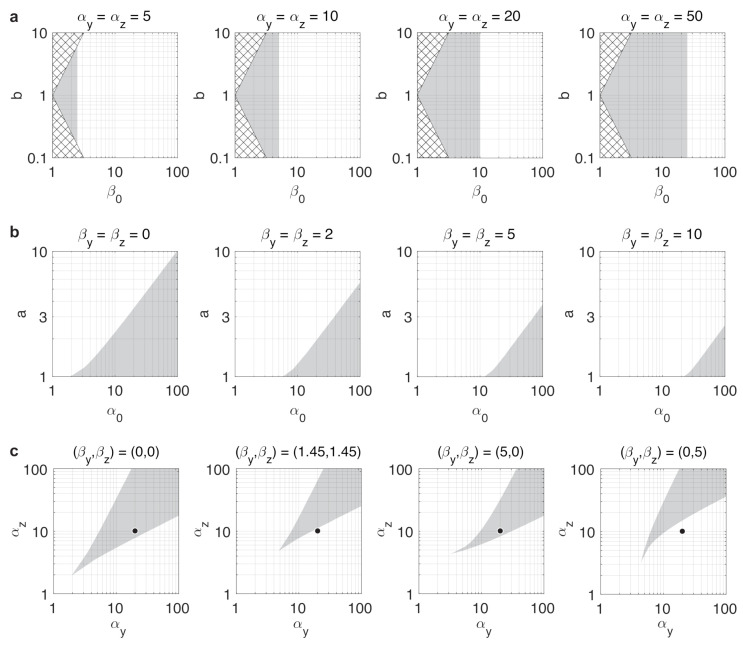
Stability properties of the toggle switch are shaped by resource competition and parameter asymmetry. Gray regions denote parameter combinations yielding bistable dynamics. Bistability/monostability was determined numerically by simulating 100 trajectories with randomly chosen initial conditions for each parameter value and clustering the endpoints. (**a**) Stability profile in case of balanced dynamics. Values of $\left(\beta_{0},b\right)$ in the checkered regions are not possible with $\beta_{y},\beta_{z}\geq 0$. (**b**) Stability profile in case of unbalanced dynamics. (**c**) Stability profile in case of unbalanced dynamics with $\beta_{0}=0$ in the first panel and $\beta_{0}=6$ in the other three panels.

For instance, we have already seen that in case of balanced toggle switches (i.e., a=1), the dynamics are bistable if $\alpha >2\beta_{0}$ [61], independent of the value of *b* (Figure 3a). In case of unbalanced switches, from (12) we expect that greater values of *a* push the dynamics towards monostability by decreasing the right-hand side of (12), whereas increasing $\alpha_{0}$ would have the opposite effect, confirmed in Figure 3b. Our results also reveal that the repressor with stronger expression can tolerate higher resource sequestration without losing bistability. For instance, assume that a>1, thus $\alpha_{y}>\alpha_{z}$ (black circles in Figure 3c). As greater values of $\beta_{0}$ increase the left-hand side of (12), we expect this change to push the dynamics towards monostability. Furthermore, keeping $\beta_{0}$ constant (representing the same overall amount of resource sequestration), while increasing *b* decreases the left-hand side, thus pushes the dynamics towards bistability, decreasing *b* has the opposite impact. These effects are confirmed and illustrated in Figure 3c: while low $\beta_{0}$ yields bistable dynamics despite the unbalancedness (first panel), increasing it evenly for both sides of the toggle switch causes a shift to monostability (second panel). Conversely, shifting the same amount of overall resource sequestration exclusively towards the side with higher production rate constant α preserves bistability (third panel), whereas allocating it to the other side has the opposite effect (fourth panel).

### 3.2. Robustness Properties

**Figure 4:**
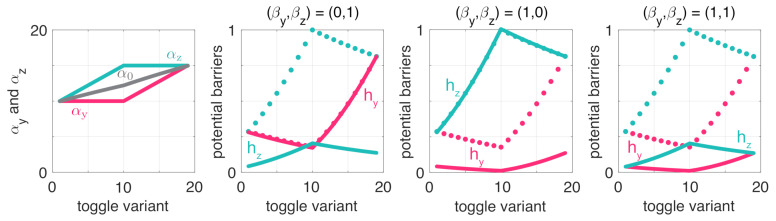
Robustness properties of the toggle switch are shaped by resource competition and parameter asymmetry. Solid lines denote the values with the indicated resource sequestration, whereas dotted lines correspond to the case when $\left(\beta_{y},\beta_{z}\right)=\left(0,0\right)$. Potential barriers are all normalized by the same factor so that the maximum across all plots is 1.

Having revealed how resource sequestration and parameter asymmetry affect the stability profile of (6), we next focus on the robustness properties of the metastable fixed points considering (8). As detailed in the previous section, according to the Eyring–Kramers formula, the mean transition time between these points is proportional to the height of the potential barriers separating them. Therefore, here we first focus on how these barriers are shaped by increased competition for shared resources and unbalancedness. To this end, consider a variety of toggle switches with different pairs of $\left(\alpha_{y},\alpha_{z}\right)$ and progressively increasing $\alpha_{0}=\sqrt{\alpha_{y}\alpha_{z}}$ (Figure 4, first panel).

To reveal the role of balancedness, assume first that $\beta_{y}=\beta_{z}=0$. Note that from a stability perspective, increasing $\alpha_{0}$ pushes the dynamics towards bistability (Figure 3); thus, it is reasonable to expect that robustness to noise also increases as the dynamics lie farther from the monostable/bistable border. While this is certainly the case for balanced switches (toggle variants #1 and #19) as the potential barriers $h_{y}$ and $h_{z}$ increase with $\alpha_{0}$ (red and green dotted lines in Figure 4), the relationship in case of unbalanced toggle switches is more nuanced (toggle variants #2–#18). In particular, as $\alpha_{0}$ increases by first increasing $\alpha_{z}$ while keeping $\alpha_{y}$ constant (toggle variants #2–#10), $h_{z}$ is indeed increasing rapidly but at the expense of $h_{y}$ decreasing (red and green dotted lines in Figure 4). Therefore, while increasing $\alpha_{0}$ in this case pushes the dynamics farther away from monostability (Figure 3), only the $x_{z}$ metastable state becomes more robust to noise, the other’s sensitivity to noise instead increases, and a similar trend can be observed when the roles are reversed (toggle variants #10–#18).

The role of resource sequestration can be analyzed similarly. As increasing $\beta_{0}$ pushes the dynamics towards monostability (Figure 3), we expect it to have a negative effect on the robustness of metastable states to noise. This is indeed the case, but parameter asymmetries also play a key role. In particular, the results in Figure 4 illustrate that while increasing $\beta_{y}$ and $\beta_{z}$ fundamentally affect $h_{y}$ and $h_{z}$, thus the robustness of $x_{y}$ and $x_{z}$ to noise, respectively, cross effects are negligible (Figure 4). That is, increasing loading on one side renders the same side more sensitive to random switchings, but leaves the other side unaffected.

Next, we focus on how the robustness of the metastable states shape the population-level composition of colonies. To this end, note that from the Eyring–Kramers formula the mean transition time $\tau_{y}$ is proportional to $\exp\left(h_{y}/\epsilon \right)$ where ϵ regulates noise intensity [85,86,87,88]. Furthermore, assuming that random $x_{y}\to x_{z}$ transitions are distributed exponentially [89] with parameter $1/\tau_{y}$ (so that the mean wait time is precisely $\tau_{y}$), and considering the doubling time $t_{d}=\ln\left(2\right)/\gamma$ where γ is the growth rate, we obtain that the probability of a random $x_{y}\to x_{z}$ switching between consecutive doublings is given by $p_{y}=1-e^{-t_{d}/\tau_{y}}$. This highlights that $p_{y}$ increases with the doubling time $t_{d}$ and decreases with $\tau_{y}$, thus increases with noise intensity and decreases with the potential barrier $h_{y}$. Similarly, we obtain that $p_{z}=1-e^{-t_{d}/\tau_{z}}$ for $x_{z}\to x_{y}$ transitions. Having uncovered how the potential barriers are shaped by the interplay between competition for shared resources and balancedness (Figure 4), we next focus on how $p_{y}$ and $p_{z}$ affect the evolution of the population-level composition of colonies by computing $q_{i}\left(\cdot \right)$ and $r_{i}\left(\cdot \right)$ from (11). To this end, from Figure 3 with $y_{i}=2y_{i}^{\prime }$ and $z_{i}=2z_{i}^{\prime }$ it follows that

$$\begin{array}{cc} q_{i}\left(y_{i}\right)= & \mathrm{Pr}\left(Y_{i}^{\prime }=y_{i}^{\prime }\;|\;Y_{0}=0\right)=\sum_{y_{i-1}=0}^{2^{i-1}}\mathrm{Pr}\left(Y_{i}^{\prime }=y_{i}^{\prime },\;Y_{i-1}=y_{i-1}\;|\;Y_{0}=0\right)\\ = & \sum_{y_{i-1}=0}^{2^{i-1}}\mathrm{Pr}\left(Y_{i}^{\prime }=y_{i}^{\prime }\;|\;Y_{i-1}=y_{i-1},\;Y_{0}=0\right)\mathrm{Pr}\left(Y_{i-1}=y_{i-1}\;|\;Y_{0}=0\right)\\ = & \sum_{y_{i-1}=0}^{2^{i-1}}\mathrm{Pr}\left(Y_{i}^{\prime }=y_{i}^{\prime }\;|\;Y_{i-1}=y_{i-1}\right)q_{i-1}\left(y_{i-1}\right),\\ r_{i}\left(y_{i}\right)= & \mathrm{Pr}\left(Y_{i}^{\prime }=y_{i}^{\prime }\;|\;Y_{0}=1\right)=\sum_{y_{i-1}=0}^{2^{i-1}}\mathrm{Pr}\left(Y_{i}^{\prime }=y_{i}^{\prime },\;Y_{i-1}=y_{i-1}\;|\;Y_{0}=1\right)\\ = & \sum_{y_{i-1}=0}^{2^{i-1}}\mathrm{Pr}\left(Y_{i}^{\prime }=y_{i}^{\prime }\;|\;Y_{i-1}=y_{i-1},\;Y_{0}=1\right)\mathrm{Pr}\left(Y_{i-1}=y_{i-1}\;|\;Y_{0}=1\right)\\ = & \sum_{y_{i-1}=0}^{2^{i-1}}\mathrm{Pr}\left(Y_{i}^{\prime }=y_{i}^{\prime }\;|\;Y_{i-1}=y_{i-1}\right)r_{i-1}\left(y_{i-1}\right), \end{array}$$

together with Pr(Yi′=yi′|Yi−1=yi−1)=∑ny=0yi−1yi−1nypymy(1−py)nyzi−1nzpzmz(1−pz)nz where $z_{i-1}=2^{i-1}-y_{i-1}$, $m_{y}=y_{i-1}-n_{y}$, $m_{z}=y_{i}^{\prime }-n_{y}$, and $n_{z}=2^{i-1}+n_{y}-y_{i}^{\prime }$ from Figure 3. Note that here we used the generalized definition of the binomial coefficients such that nk=0 if k<0 or if k>n. Therefore, with the initial conditions $q_{0}\left(0\right)=r_{0}\left(1\right)=1$ and $q_{0}\left(1\right)=r_{0}\left(0\right)=0$ we can recursively compute $q_{i}\left(y_{i}\right)$ and $r_{i}\left(y_{i}\right)$.

**Figure 5:**
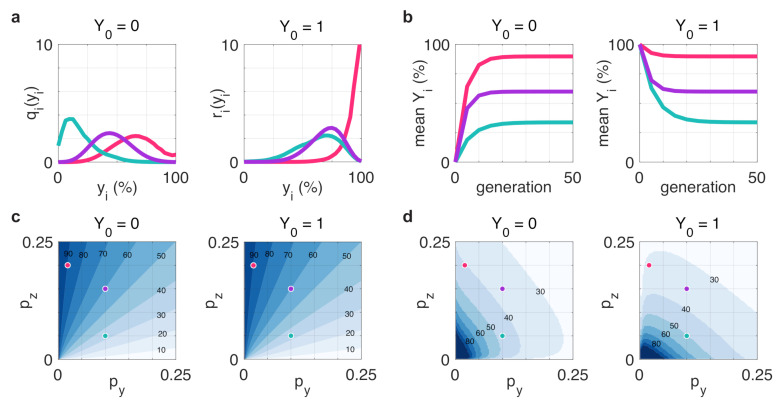
Evolution of population-level distribution of cells starting from a *z*-dominant and *y*-dominant initial cell ($Y_{0}=0$ and $Y_{0}=1$, respectively). (**a**) Population-level distribution of cells in the *y*-dominant state in generation 5. (**b**) The average number of cells in the *y*-dominant state after successive doublings. (**c**) The mean percentage of the population in the *y*-dominant state at equilibrium. (**d**) The number of generations required to (approximately, within 0.1% range) reach the steady state distribution. Simulation parameters: $\left(p_{y},p_{z}\right)=\left(0.02,0.2\right)$, $\left(p_{y},p_{z}\right)=\left(0.1,0.05\right)$, $\left(p_{y},p_{z}\right)=\left(0.1,0.15\right)$ for red, green, and purple, respectively.

This result reveals that depending on the transition probabilities $p_{y}$ and $p_{z}$, after only a few generations the population-level profile of steady-state distribution can fundamentally differ from the state of the initial seed cell (e.g., red in Figure 5a starting from $Y_{0}=1$), especially if the distribution mean at the steady state is substantially different from the initial state (Figure 5b). Importantly, the expected composition of the population at steady state does not depend on the state of the initial seed cell (Figure 5c), but the speed at which this state is reached does (Figure 5d).

### 3.3. Balancing via Optimized Competition

The balancedness of the toggle switch not only fundamentally affects its stability and robustness properties, as we have uncovered in this paper (Figure 3, Figure 4 and Figure 5), it is also crucial when the toggle switch is utilized as a “digital comparator” to optimize cellular performance [67]. We have already highlighted that carefully chosen resource competition can restore bistability (Figure 4c), thus here we explore how it can be leveraged to increase balancedness.

**Figure 6:**
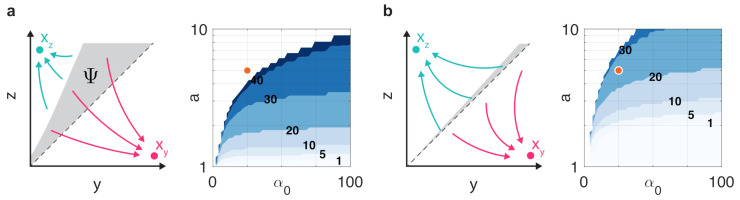
Resource competition can be leveraged to increase the balancedness of the toggle switch (uncolored regions correspond to parameter combinations that yield monostable dynamics). Contour values represent $e_{\Psi }$ in percentages. (**a**) In the absence of resource competition (i.e., $\beta_{y}=\beta_{z}=0$), differences between $\alpha_{y}$ and $\alpha_{z}$ lead to significant error $e_{\Psi }$. (**b**) By the optimal choice of $\left(\beta_{y},\beta_{z}\right)$ the error $e_{\Psi }$ is greatly reduced.

To illustrate this, first consider the case when resource competition is neglected (i.e., $\beta_{y}=\beta_{z}=0$) and the toggle switch is balanced (i.e., $\alpha_{y}=\alpha_{z}$). In this case, trajectories converge to $x_{y}$ and $x_{z}$ if y(0)>z(0) and if y(0)<z(0), respectively, that is, to the fixed point that corresponds to the dominant initial coordinate. In case of an unbalanced toggle switch (i.e., $\alpha_{y}\neq \alpha_{z}$), this is not true anymore: for instance, if $\alpha_{y}>\alpha_{z}$ then some initial conditions where y(0)<z(0) will yield trajectories that converge erroneously to $x_{y}$ (gray region in Figure 6a). To measure this effect, for the initial condition $\left(y_{0},z_{0}\right)$ let $\left(y,z\right)\to \left(y_{\infty },z_{\infty }\right)$ as t→∞ and define $\Psi =\left\{\left(y_{0},z_{0}\right)\;|\;\left(y_{0}-z_{0}\right)\left(y_{\infty }-z_{\infty }\right)<0\right\}$, that is, the set of initial conditions where the initial and final dominant coordinates are different (Figure 6a). To measure the size of this region, define $e_{\Psi }=\frac{1}{\alpha_{y}\alpha_{z}}{\;\int \int \;}_{\Psi }dA$, so that $e_{\Psi }$ characterizes the fraction of the rectangle $\left[0,\alpha_{y}\right]\times \left[0,\alpha_{z}\right]$ with incorrect initial/final state pairings (Figure 6a). In particular, $e_{\Psi }=0$ in case of balanced switches and $e_{\Psi }$ increases as the toggle gets increasingly more unbalanced. The data in Figure 6a confirm that while $e_{\Psi }$ increases with unbalancedness, it stays fairly constant for a given level of unbalancedness above a certain threshold value of $\alpha_{0}=\sqrt{\alpha_{y}\alpha_{z}}$.

Importantly, unbalancedness due to $\alpha_{y}\neq \alpha_{z}$ can be mitigated by carefully selecting $\beta_{y}$ and $\beta_{z}$, e.g., via the introduction of decoy sites [65]. This is illustrated in Figure 6b where the optimal choice of $\left(\beta_{y},\beta_{z}\right)$ significantly reduces the error $e_{\Psi }$: if $1\leq a=\alpha_{y}/\alpha_{z}\leq 2$ the error decreases from 20% (Figure 6a) to less than 1% (Figure 6b), rendering the toggle switch almost perfectly balanced. Additionally, this optimal loading of the toggle switch also expands the range of $\left(\alpha_{0},a\right)$ pairs that yield bistable dynamics, rendering previously monostable dynamics bistable (e.g., orange circle in Figure 6).

### 3.4. Context Effects

Here, we reveal how competition for shared resources originating in the genetic context of the toggle switch shapes its stability and robustness properties. To this end, let $\beta_{c}$ capture the resource sequestration of modules other than the toggle switch (its context), yielding the dynamics

$$\dot{y}=\frac{\frac{\alpha_{y}}{1+z^{2}}}{1+\frac{\beta_{y}}{1+z^{2}}+\frac{\beta_{z}}{1+y^{2}}+\beta_{c}}-y,\;\dot{z}=\frac{\frac{\alpha_{z}}{1+y^{2}}}{1+\frac{\beta_{y}}{1+z^{2}}+\frac{\beta_{z}}{1+y^{2}}+\beta_{c}}-z.$$

Importantly, with $\alpha_{w}^{\prime }=\alpha_{w}/\left(1+\beta_{c}\right)$ and $\beta_{w}^{\prime }=\beta_{w}/\left(1+\beta_{c}\right)$ for w∈{y,z} we obtain the dynamics in (6) with these rescaled parameters instead of the original $\alpha_{w}$ and $\beta_{w}$. Thus, the results presented in this paper can be applied in a straightforward manner even in the presence of loading from the context of the toggle switch.

**Figure 7:**
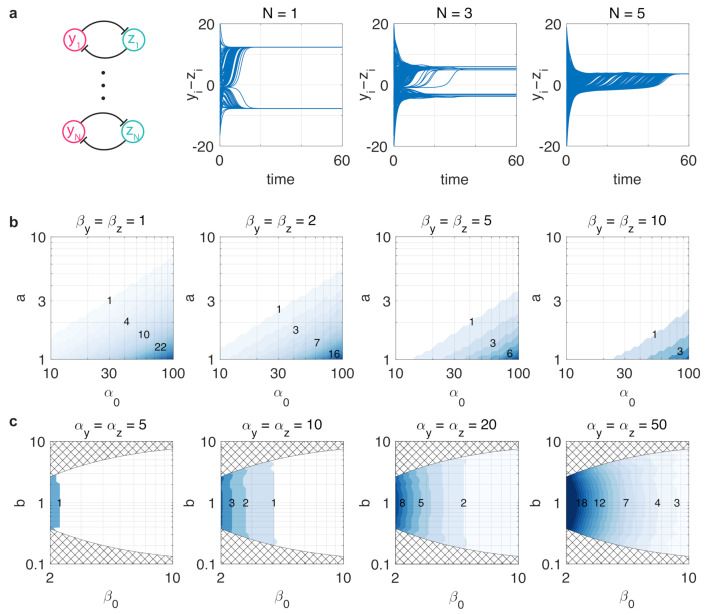
Resource competition arising in the genetic context of toggle switches can fundamentally alter their behavior. (**a**) Bistable toggle switches can render each other monostable due to increased resource sequestration. Simulation parameters are $\left(\alpha_{y},\alpha_{z}\right)=\left(25,20\right)$ and $\left(\beta_{y},\beta_{z}\right)=\left(1,1.5\right)$ with 100 randomly selected initial conditions from ${\left[0,\alpha_{0}\right]}^{2}$. (**b**) The critical number $N_{crit}$ in case of unbalanced realizations (i.e., $\alpha_{y}\neq \alpha_{z}$). (**c**) The critical number $N_{crit}$ in case of balanced realizations (i.e., $\alpha_{y}=\alpha_{z}$). Values of $\left(\beta_{0},b\right)$ in the checkered region are not possible with $\beta_{y},\beta_{z}\geq 0$.

To illustrate the detrimental effects of such competition, consider the collective behavior of *N* toggle switches, given by the dynamics

$${\dot{y}}_{i}=\frac{\frac{\alpha_{y,i}}{1+z_{i}^{2}}}{1+\frac{\beta_{y,i}}{1+z_{i}^{2}}+\frac{\beta_{z,i}}{1+y_{i}^{2}}+\beta_{c,i}}-y_{i},\;{\dot{z}}_{i}=\frac{\frac{\alpha_{z,i}}{1+y_{i}^{2}}}{1+\frac{\beta_{y,i}}{1+z_{i}^{2}}+\frac{\beta_{z,i}}{1+y_{i}^{2}}+\beta_{c,i}}-z_{i},$$

where $\beta_{c,i}=\sum_{j\neq i}\left(\frac{\beta_{y,i}}{1+z_{i}^{2}}+\frac{\beta_{z,i}}{1+y_{i}^{2}}\right)$ for i=1,2,⋯,N. While a single toggle switch alone may display a bistable stability profile, the addition of further switches decreases the separation of the two stable fixed points (Figure 1), eventually leading to the collectively monostable dynamics of individually bistable toggle switches (Figure 7a) as a result of additional loading from each other [61].

To reveal the role that parameter asymmetry and resource competition play in the above phenomenon, consider the simulation data in Figure 7b,c illustrating the critical number $N_{crit}$ of identical toggle switches (i.e., $\alpha_{w}=\alpha_{w,i}$ and $\beta_{w}=\beta_{w,i}$ for w∈{y,z} and $i=1,2,\cdots ,N_{crit}$) such that one more unit would render the collective behavior monostable. These results reveal two key findings, which follow from the data in Figure 4a,b. First, $N_{crit}$ increases with $\alpha_{0}=\sqrt{\alpha_{y}\alpha_{z}}$ and decreases with $\beta_{0}=\sqrt{\left(1+\beta_{y}\right)\left(1+\beta_{z}\right)}$. Second, while asymmetry in the parameters measuring resource loading via $\beta_{y}$ and $\beta_{z}$ captured by $b=\left(1+\beta_{y}\right)/\left(1+\beta_{z}\right)$ does not have any appreciable effect, the balancedness of the toggle switch (i.e., $a=\alpha_{y}/\alpha_{z}\approx 1$) is crucial, as increasing the difference between $\alpha_{y}$ and $\alpha_{z}$ significantly decreases $N_{crit}$. These results once again underscore that unbalancedness exacerbates the detrimental effects of resource competition. Naturally, as the number of toggle switches increases and approaches $N_{crit}$, additional resource competition also yields reduced robustness to noise, following directly from Figure 4.

## 4. Discussion

Given that genetic modules display context-dependent behavior [9,25,28], predictive and quantitative models play a fundamental role in the development of complex genetic circuits [36,37]. One major source coupling the behavior of seemingly unconnected components emerges due to the limited availability of shared cellular resources, thus introducing the “bioenergetic cost” of genes due to their existence and expression [93]. As recent experimental developments enable the precise characterization and separation of this cost into expenses that cells incur at various levels [94,95], these high-throughput technologies illuminate the inner workings of cells at the part-level. As a result, leveraging quantitative modeling and formal analytic tools offer promising avenues for aiding the rational design of synthetic gene circuits.

Therefore, in this paper we focused on revealing how competition for shared cellular resources affects the stability and robustness properties of one of the most widely used genetic modules: the toggle switch [60]. This core building block plays a central role in a vast array of both natural and synthetic systems. One prominent example is checkpoint control enabling the division of complex tasks into independent sub-tasks [96], allowing cells to respond to a wide variety of input signals [97] influencing a diverse set of cellular processes [98,99]. Given their central role, understanding how fundamental dynamic properties of the toggle switch depend on tunable biophysical parameters in real-world applications is thus of considerable interest to promote modularity and predictable system-level performance.

To this end, our combined analytical/numerical approach uncovered explicit guidelines aiding the design and tuning of genetic switches in a rational fashion, even in the presence of competition for shared transcriptional and translational resources. For instance, we revealed that while greater protein expression rates push the dynamics towards bistability and yield increased robustness to noise, resource competition has the opposite effect. Furthermore, our findings highlight that parameter asymmetries play a crucial role in establishing stability and robustness properties: not only can they exacerbate detrimental effects of resource competition, but also restore bistability and balancedness when carefully optimized.

To obtain the results presented in this paper, we explicitly modeled the scarcity of shared resources and the resulting coupling phenomena, both between genes of the toggle switch and arising due to its genetic context. The reduced order model underpinning our findings offers a realistic approximation of the dynamics of the switch as it considers parameter asymmetries and the model assumptions lead to accurate experimental predictions in vitro and in vivo [26,28]. As the parameters all possess clear physical interpretations and correspond to easily tunable properties of standard genetic parts, the design guidelines we uncovered are directly translatable to experimental considerations. For instance, α can be tuned via ribosome binding site engineering [68], β via the introduction of decoy sites [65], and $\beta_{c}$ via the expression of heterologous proteins [25,26].

The results presented here are complemented by recent efforts to mitigate the adverse effects of competition for shared cellular resources, for instance, by decoupling resource-coupled gene expression [100], upregulating ribosome production reacting to increased metabolic burden [101], and splitting up multicomponent genetic systems into smaller subcomponents distributed among multiple collaborative cell strains [102]. Additionally, as integrative models [10,32,33,34,40] can accurately capture the bidirectional coupling between genetic circuits and the host harboring them, they offer unique and invaluable insights for the synthesis of large-scale biocircuits, for instance, by revealing how cell proliferation and gene expression affect one another. Our findings together with these tools thus offer promising opportunities for the rational and context-aware design of genetic switches relying on carefully characterized parts [103]. Therefore, we expect the key findings presented in this paper to be incorporated into the computer-aided fabrication of large-scale synthetic circuits [96,104,105], among other effects of context-dependence [9,15,22,23,24].

## Figures and Tables

**Table 1:** Typical range of parameter values.

Symbol	Meaning	Typical Value	Unit	Reference
κ	RNAP dissociation constant	1	μM	[22,26]
*k*	ribosome dissociation constant	10	μM	[26,81]
*K*	repressor dissociation constant	0.1	nM	[82]
*D*	DNA concentration	100–1000	nM	[26,83]
$\lambda^{\mathrm{TX}}$	transcriptional rate constant	100	1/h	[26,81]
$\lambda^{\mathrm{TL}}$	translational rate constant	1000	1/h	[26,81]
δ	mRNA decay rate constant	10	1/h	[84]

## Supplementary Materials

The MATLAB scripts required for obtaining the simulation data are available online at https://github.com/netbio-lab/unbalanced-toggle.git. All data were obtained using MATLAB R2020b, figures were prepared using Adobe Illustrator 2021.

## Appendix A. Stability Profile of the Unbalanced Toggle Switch

**Theorem** **A1** (Adopted from [64]). *Fix a≥1 and define $\bar{w}=a+\sqrt{a^{2}-1}\geq 1$. Define*

(A1) $$g_{1}\left(z\right)=\frac{\alpha_{z}}{\beta_{y}}\frac{1}{1+z^{2}},\;g_{2}\left(z\right)=\frac{1}{\beta_{y}}\left(1+\frac{\beta_{z}}{1+z^{2}}\right),\;g\left(y,z\right)=\frac{g_{1}\left(z\right)}{g_{2}\left(z\right)+\frac{1}{\left(1+y^{2}\right)}},$$

*and introduce ${\underset{\bar{}}{G}}_{1}\left(z\right)=0$, ${\bar{G}}_{1}\left(z\right)=\alpha_{z}$, together with ${\underset{\bar{}}{G}}_{k+1}\left(z\right)=g\left({\underset{\bar{}}{G}}_{k}\left(z\right),z\right)$ and ${\bar{G}}_{k+1}\left(z\right)=g\left({\bar{G}}_{k}\left(z\right),z\right)$ for k=1,2,⋯, and let $\underset{\bar{}}{G}\left(z\right)=\lim_{k\to \infty }{\underset{\bar{}}{G}}_{k}\left(z\right)$ and $\bar{G}\left(z\right)=\lim_{k\to \infty }{\bar{G}}_{k}\left(z\right)$. The dynamics (6) are bistable if $\underset{\bar{}}{G}\left(1\right)\geq \bar{w}$ and monostable if $\bar{G}\left(1\right)\leq \bar{w}$.*

From Theorem A1 it follows that sufficient conditions for bistability and monostability of (6) are

(A2) $$\frac{g_{1}\left(1\right)}{g_{2}\left(1\right)+1}\geq a+\sqrt{a^{2}-1},\;\frac{g_{1}\left(1\right)}{g_{2}\left(1\right)}\leq a+\sqrt{a^{2}-1},$$

respectively. As $\alpha_{z}=\alpha_{0}/\sqrt{a}$, the first condition in (A2) is equivalent to

$$\frac{\alpha_{0}}{\sqrt{a}+\sqrt{a-\frac{1}{a}}}\geq 2+2\beta_{y}+\beta_{z}.$$

Furthermore, as $b=\left(1+\beta_{y}\right)/\left(1+\beta_{z}\right)$ and $\beta_{0}=\sqrt{\left(1+\beta_{y}\right)\left(1+\beta_{z}\right)}$, we have that

$$2+2\beta_{y}+\beta_{z}\leq 2\left(1+\beta_{y}\right)+\left(1+\beta_{z}\right)=2\beta_{0}\sqrt{b}+\frac{\beta_{0}}{\sqrt{b}}=\beta_{0}\frac{1+2\sqrt{b}}{\sqrt{b}}.$$

Therefore, if

$$\frac{\alpha_{0}}{\sqrt{a}+\sqrt{a-\frac{1}{a}}}\geq \beta_{0}\frac{1+2\sqrt{b}}{\sqrt{b}}$$

then the first condition in (A2) is satisfied, thus (6) is bistable. Similarly, the second condition in (A2) is equivalent to

$$\frac{\alpha_{0}}{\sqrt{a}+\sqrt{a-\frac{1}{a}}}\leq 2+\beta_{z}=1+\left(1+\beta_{z}\right)=1+\frac{\beta_{0}}{\sqrt{b}},$$

thus (6) is monostable if

$$\frac{\alpha_{0}}{\sqrt{a}+\sqrt{a-\frac{1}{a}}}\leq 1+\frac{\beta_{0}}{\sqrt{b}}.$$

## Appendix B. Computation of the Potential Landscape and the Potential Barriers

While (9) details how the quasi-potential is calculated along system trajectories, the initial value of V(y,z) is not specified at the beginning of each trajectory. As detailed in [90], this lets us satisfy two fundamental properties of the potential landscape: (i) trajectories converging to identical fixed points have the same potential at their endpoints and (ii) trajectories starting sufficiently close have the same initial potential at their origin. With this, we can calculate the potential landscape and the barriers $h_{y}$ and $h_{z}$ separating the metastable fixed points $x_{y}$ and $x_{z}$ in case of bistable dynamics as follows:

1. locate the unique unstable and the two stable fixed points of (6);
2. create a set of initial conditions in the range $\left(y,z\right)\in \left[\alpha_{y},\alpha_{z}\right]$;
3. compute the potential decrease along system trajectories starting from the above initial points according to (9);
4. using k-means clustering, partition the endpoints of these trajectories into two clusters;
5. having identified the two regions of convergence ($\Omega_{y}$ and $\Omega_{z}$), adjust the initial potentials in both of them so that trajectories converging to the same stable fixed point have the same end potential;
6. adjust the initial potentials alongside the border separating $\Omega_{y}$ and $\Omega_{z}$ so that trajectories starting close but on different sides share the same potential; and
7. calculate the potential barriers $h_{y}$ and $h_{z}$ according to (10).
